# “Real time in different spacetime”: a phenomenology of networked music performance

**DOI:** 10.3389/fpsyg.2026.1855693

**Published:** 2026-07-03

**Authors:** Seth Adams

**Affiliations:** University of Massachusetts Lowell, Lowell, MA, United States

**Keywords:** Actor-Network Theory (ANT), improvisation (music), latency, networked music performance (NMP), phenomenology, telepresence

## Abstract

What counts as “real time” when musicians perform together across distance? Networked Music Performance (NMP) challenges conventional assumptions about simultaneity, presence, and ensemble by making perceptible the relations that co-located music performance often leaves tacit. This study presents a phenomenological analysis of interviews with 15 experienced NMP practitioners, yielding three primary themes: intimacy at a distance, improvisation, and practical hope for the medium. The central essence of the experience was a felt sense of expanded musical possibility across physical distance. Participants described feelings of intimacy as shaped by studio-quality sound, domestic comfort, and the simultaneous chance for globe-spanning connections. They also described adapting to latency through flexible temporal strategies and musical creativity. Practical hope for the medium appeared in participants' pursuit of appropriate tools, recruitment and onboarding of newcomers, and a willingness to directly confront skeptics of NMP's potential. Interpreted through Actor-Network Theory, these accounts suggest that NMP is assembled through relations among musicians, instruments, tools, software, and infrastructures, all of which enter directly into the lived musical event. Findings position NMP as a contingent but meaningful temporal ecology in which multiple presents can coexist and musical relation remains possible under conditions of network mediation.

## Introduction

Networked Music Performance (NMP) is a form of interpersonal coordination in which participants continuously adapt their actions in relation to one another and to constraints emerging from the interplay of musical and network conditions. Research in both sport and music has shown that such coordination is both behavioral and phenomenological, involving timing, adjustment, and shared or divergent apprehensions of the joint activity (Sève et al., 2013; Seifert et al., 2017). NMP offers a particularly revealing case of ensemble performance, since musicians must still achieve coordinated timing and a cohesive shared sound while adapting to technologically mediated conditions.

Early studies into NMP demonstrated that audio delay (i.e., latency) disrupts musical coordination in predictable ways while also showing that musicians can adapt using a range of strategies (Schuett, 2002; Chafe et al., 2004). Subsequent work suggested that latency thresholds are not fixed, but vary according to musical context, rhythmic content and performer experience (Carôt et al., 2009; Chafe et al., 2010). Parallel lines of inquiry examined the use of networked systems for music teaching and learning, with explicitly low-latency platforms shown to support forms of remote instruction more effectively than standard videoconferencing tools (Riley et al., 2016). More recently, Tsioutas et al. (2025), working in a controlled laboratory environment with 11 pairs of musicians, found that performers could synchronize at one-way delays of up to 40 ms, that audio quality could be reduced substantially without significant quality-of-experience loss, and that musicians relied more on audio than video for synchronization.

Research into NMP has also moved toward examining the quality of communication and artistic experience. Iorwerth and Knox's (2024) Playing Together, Apart Framework (PTAF) proposes a standardized means of considering communication between musicians in NMP. The framework is based on duo musicians working in informal NMP situations and shows the audio and video communication paths, along with the influences on both the transmission and reception elements of the communication chain. Rhythmic content, the expertise and experience of the musicians when dividing attention, the use of video, and the socio-emotional and professional relationships between musicians all emerge as salient under NMP conditions.

Related research has also begun to examine mediated presence more directly. In a comparison of in-person synchronous and remote asynchronous singing, Fancourt and Steptoe (2019) found greater reported social presence in the asynchronous virtual choir, alongside greater use of emotional regulation strategies in the live choir, a result that ran counter to their expectation that the two measures would positively correlate. In a multiple case study of remote music therapy during the COVID-19 pandemic, Schmid et al. (2021) identified mutual presence as one of four central themes, alongside embodiment, space and time, and mediation, concluding that online musicking still carried therapeutic force for residents and carers. Together, these studies suggest that network-mediated musical environments can generate meaningful forms of presence and relation.

Lind et al. (2025) move the conversation further into the aesthetic dimensions of telematic performance (a term for NMP which extends beyond music to include multimedia and intermedia time-based arts). In their evaluation of a latency-accepting approach to intercontinental telematic composition and performance, latency is treated as an artistic parameter. Drawing on McLuhan's (1964) concepts of *extension* and *amputation*, Lind and colleagues show how the telematic environment reshapes traditional musical practices. Composers, in their account, tend to perceive the telematic environment as an *extension* through which new forms of temporal and spatial exploration emerge, while performers emphasize *amputations*, particularly the lack of real-time audience feedback and physical interaction. Their study also highlights the importance of synchronized notation and visual cues in maintaining ensemble cohesion and facilitating creative expression in telematic contexts. This recent work is especially useful because it makes plain that NMP does not simply transmit an existing performance situation across distance. It reorganizes the situation, simultaneously opening certain possibilities while constraining others.

“Videoconferencing technology now supports a wide range of music ventures. Challenges of latency, audio and video quality, presence (i.e., embodiment), and the resulting new online music teaching approaches have yet to be explored in depth through formal research.” (Lisboa et al., 2022; p. 499)

This assessment from a chapter in *The Oxford Handbook of Music Performance* captured a moment in which the challenges named were still largely underexamined. Subsequent work has begun to address them directly, particularly in relation to synchronization, communication, and aesthetic practice. What remains less fully described, however, is the essential, subjectively lived experience of NMP for musicians accustomed to the medium: how participants perceive and confront latency, fidelity, and telepresence; how they understand presence when making music together apart; and how they experience the peculiar mixture of separation and connection that NMP affords.

As a phenomenological study, this project describes the essential structure of NMP as lived by a meaningful sample of experienced practitioners. It therefore attends closely to participants' first-person descriptions and reduces them toward invariant meanings and structures of experience (Moustakas, 1994). Tsioutas et al. (2025) have clarified conditions of delay, quality, and synchronization, Iorwerth and Knox (2024) have clarified the communication chain within informal NMP, and Lind et al. (2025) have clarified the artistic possibilities and losses of latency-accepting telematic work. The remaining question is how these conditions are synthesized in experience: how latency, fidelity, telepresence, distance, and related actors become part of a coherent musical practice. The Results section first addresses this question at the level of lived description; the Discussion then turns to Actor-Network Theory (ANT) to consider how those experiences were assembled.

In ANT, action is mediated by networks of human and nonhuman actors rather than originating in any single source (Latour, 2005). ANT is especially helpful for the present study because NMP is always multiply mediated (Born, 2005). Although Born would likely note that this is true of all music-making, in co-located performance many mediations typically recede into familiarity. In NMP, by contrast, mediations are often top of mind for practitioners. Latency, routing, headphone mix, microphone selection, bandwidth, and regional network quality can all shape the same musical moment in ways performers must actively confront. ANT therefore provides a useful framework for understanding how musical connection is assembled under networked conditions, and why it is experienced as variable and irreducibly mediated.

## Materials and methods

This qualitative study used Moustakas's transcendental phenomenological approach to examine the lived experience of NMP for musicians accustomed to the medium. The method was selected because the study sought to describe the essential structure of a form of musical practice shaped by latency, technological mediation, and distributed presence. In contrast to research centered primarily on technical thresholds or communication pathways, this approach allowed the analysis to remain grounded in participants' first-person accounts of how the medium is lived and how their performances are assembled.

### Participants

Using purposeful sampling (Creswell and Poth, 2024), I recruited 17 experienced users of NMP. Eligibility criteria included a minimum age of 18 years, a minimum of 25 estimated lifetime hours spent practicing dyad and/or ensemble NMP, and no more than 1 month since the participant's most recent NMP session. These criteria were designed to ensure that participants could describe the phenomenon from sustained and recent experience.

Participants were recruited through my professional network as well as through online forums and groups for popular NMP applications. 17 interviews were conducted. Two interviews were excluded after preliminary review because they yielded insufficient phenomenological detail for textural-structural analysis. The final sample therefore consisted of 15 participants. 12 participants were based in the United States, two in Europe, and one in South America. The study was approved by the relevant institutional review board, and all participants provided informed consent prior to participation. All names used in this manuscript are approved pseudonyms.

### Procedure

Before interviewing participants, I engaged in reflexive bracketing, or epoché, to try and suspend prior assumptions and experiences related to NMP and remain open to the phenomenon as described by participants (Moustakas, 1994). Because I had prior research and practical experience with NMP, this process involved explicitly setting aside evaluative judgments about platforms, practices, and my own earlier work. During interviews, I therefore avoided introducing personal opinions or comparisons and focused on eliciting participants' first-person descriptions of their experiences.

Interviews were conducted and recorded via Zoom (*n* = 13) or, by participant request, FarPlay (*n* = 2). Zoom was my preferred platform because of its ubiquity and because it allowed me to avoid the appearance of favoritism toward any one NMP software application. I conducted interviews on FarPlay only in the two cases when participants requested it by name. Other NMP softwares used by participants in their regular practice include JackTrip, Soundjack, Jamulus, and SonoBus.

Interviews lasted between 52 and 115 min, with a total dataset of 1,236 min (20 h, 36 min). Interviews began with an exercise designed to orient participants toward a specific lived experience and away from generalized opinion, consistent with Moustakas's (1994) emphasis on describing the phenomenon as concretely experienced. Participants were asked in advance to select an audio or multimedia recording of a previous NMP experience. For each interview, we listened to no more than 5 min of a recording, followed by a talk-aloud in which participants described their personal recollection of and reflection on the experience.

The subsequent interview protocol focused on participants' descriptions of NMP sessions, challenges they had experienced, relationships with other musicians with whom they had made music remotely, their joys, and their favorite benefits of the medium. Because phenomenological inquiry overlaps interview and analysis, questions were posed reflexively to clarify descriptions and develop a more fully textured apprehension of the phenomenon under study. Recordings were transcribed using a combination of computer-assisted and manual means. Zoom's built-in transcription tools were used where applicable, and all transcripts were reviewed and corrected manually. Otter.ai (Otter.ai, Inc., Mountain View, CA, USA) was also used as part of the transcription workflow.

### Analysis

Analysis was guided by Moustakas's (1994) transcendental phenomenological method. Through horizonalization, each statement about the phenomenon was initially treated as a potential horizon of experience. Irrelevant and repetitive statements were removed, leaving invariant constituents that captured the essential qualities of participants' descriptions. Through imaginative variation, these constituents were examined across differing conditions and perspectives in order to identify the structural features that give rise to the experience. These were then clustered into themes and subthemes representing shared structures of the experience. While grounded in transcendental phenomenology, analysis incorporated pragmatic qualitative procedures to support the organization and comparison of experiential meanings across a large data set.

The analysis was further refined through negative case analysis and repeated comparison across participants. Member checking was conducted by sharing findings with participants, who confirmed their accuracy and resonance with lived experience. Throughout, the analytic aim remained oriented toward description of lived experience rather than causal explanation, with a focus on articulating the essential structure of NMP as encountered by experienced practitioners.

## Results

**Figure 1:**
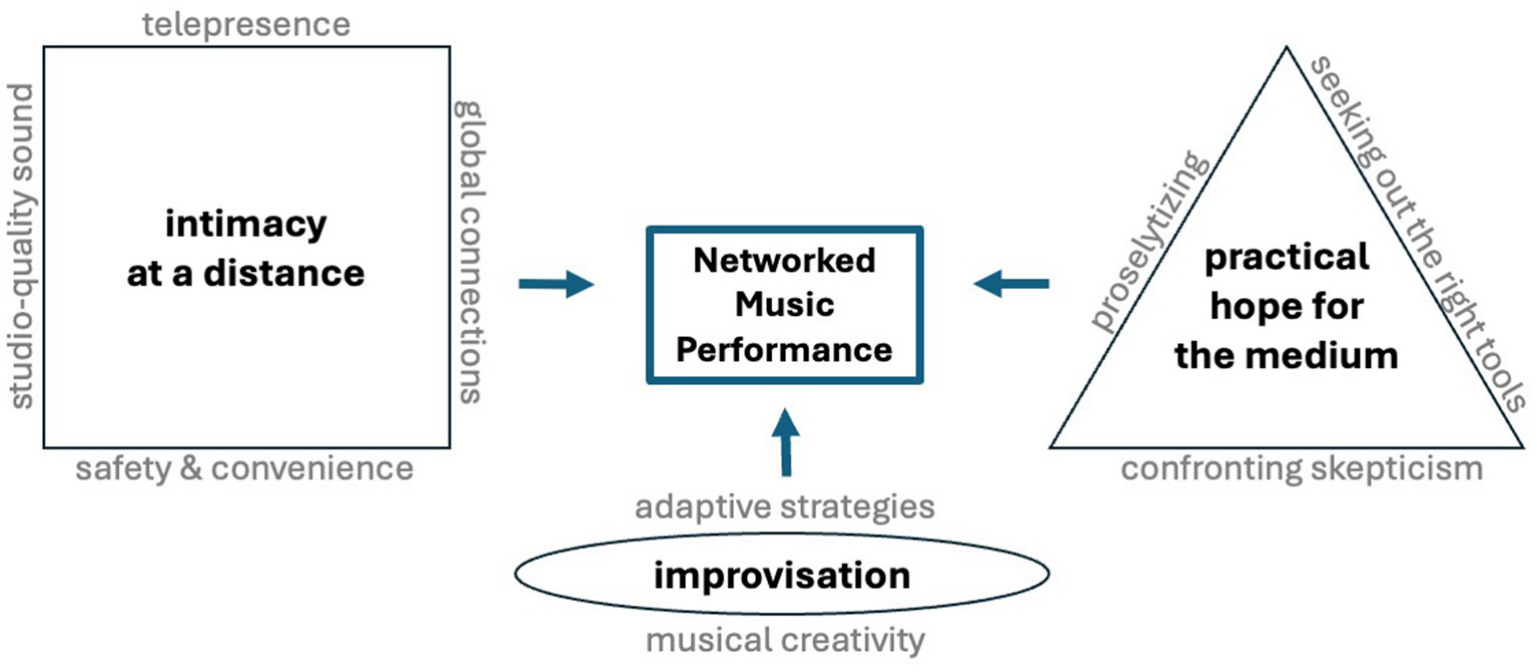
Visual representation of the three main themes of the study and their nine subthemes.

Phenomenological analysis of participants' descriptions revealed three primary themes that together describe the structure of the experience: intimacy at a distance, improvisation, and practical hope for the medium. Figure 1 provides a visual representation of themes and subthemes as identified through phenomenological analysis; it is intended as a descriptive aid rather than a conceptual model. Together, these themes articulate the structure of the lived experience of NMP as felt in private music lessons, chamber ensemble sessions, jazz collaborations, open improvisations, and larger-scale, geographically distributed electroacoustic projects. Across all 15 interviews, participants described a distinctive mode of musical experience shaped by inherent latency, sonic detail, software affordances, prior relationships, musical material, and multiple embodied settings.

### Theme 1: intimacy at a distance

Participants described NMP as fostering simultaneous perceptions of distance and intimacy. Distance presented as combinations of temporal distance (audio delay/latency), geographic distance, and/or cultural distance. Intimacy, meanwhile, related to the physical location of the individual (typically in the comfort and convenience of home) and the exquisite sonic detail possible through studio-quality microphones, headphones, and related tools. This first theme was the most prevalent across participants, and subsumed four experiential subthemes: telepresence, studio-quality sound, safety and convenience, and global connections.

#### Telepresence

I asked participants directly whether they had felt a sense of telepresence during NMP sessions, providing them with the definition,

“a state in which a participant loses their sense of locality and feels present in a virtual environment.” (paraphrased from Sheridan, 1996)

When asked this question, Declan drew a stark contrast with another act normally done only in person:

“As far as I know, there's no way of having remote sex that is anywhere close to the real thing. I'm no expert in that field, but you can't have that feeling of togetherness that having sex can bring without actually being together, right? … And I think the shocking thing about low latency audio is that this feeling that is so precious, that I *thought* you could only get by being in the same room, I've actually really had that feeling just as much [through NMP] as I would have in a room together.”

Though uncommonly vivid, Declan's account of NMP's potential for a feeling of telepresence was shared by many. John answered the same question bluntly: “If it's like a yes or no question: Yes, that's happening. You forget that you are there.” Joan described a similar effect: “It does pretty instantly melt away. And it does feel connected, especially once you've gotten used to this (medium).” Jocelyn was more circumspect: “I don't think that I feel that so much, because in my case, I'm playing multiple instruments… (the feeling) sort of comes and goes. It's not like, consistently, I'm *there*.” Jocelyn's description suggests that telepresence was not experienced as a stable condition but as something that could fluctuate depending on role, task load, and the way attention was distributed in a session.

Participants also described limits to the experience of telepresence; there was widespread agreement on the existence of a perceptual gap between the experience of NMP and that of traditional, co-located music performance. Speaking of a live-streamed, distributed NMP performance, Jordan noted with regret, “You have no idea how the audience hears it.” John, despite speaking confidently about personal feelings of telepresence, also lamented the limits of NMP: “I make use of it and I enjoy it but at the same time it's so different from just being in the same room with another human being, (which is) a much fuller experience.”

Other limits described included the inability to play certain fast-tempo selections, the inability to share food or drink together, and difficulty in communicating “warmth and emotions when you're only on the screen,” as Bella put it. Even Declan, who was almost relentlessly positive about NMP technology, conceded limitations relative to ensemble size when using peer-to-peer (P2P) networking, in which network load increases for all participants with each additional musician.

Some participants described telepresence as occupying a third, virtual space that was neither fully here nor fully there. Isaac tended to imagine such a third space as a physical room: “My concept of the virtual space that you and I are co-occupying right now: I literally imagine a room that is as large as the acoustic delay is, equivalent to the milliseconds of latency (displayed on screen in the NMP software).” More elaborately, Carter enthused about the possibility of digitally modeled acoustic environments:

“Something we've learned how to do better is the sound design on a particular locale. (For example), if we want to join in a grain silo. I've never played in a grain silo. But you can (digitally simulate such a space) and actually get the acoustics right. Right down to the level of detail of like, ‘I hear my remote partner over here, and the other remote partner over here, and if I turn my head, the perspective is correct.' And we're in a grain silo! That's now possible.”

These accounts indicate that telepresence in NMP is experienced not uniformly, but as a contingent sense of shared space shaped by musical activity, attention, and technological conditions.

#### Studio-quality sound

Participants often described the quality, character, and detail of transmitted sound as central to the experience of NMP, and many were deeply attentive to the equipment involved. They often used high-quality microphones, preamps, and USB-connected audio interfaces. Microphones in particular were described as having a strong effect on the quality of the auditory experience. Kristin, for example, explained in pedagogical terms the importance of using a flat-response condenser microphone:

“It's amazing how many voice teachers do not know that not all microphones are actually capturing the frequency spectrum equally… I have no problem with people *recording* their voices using fancy studio microphones with vocal sweeteners, right? But because I'm a voice teacher, I want to make sure that I'm hearing my students with fidelity, so a flat frequency response is very important, (and so is) not having proximity effects, where if they get close to the mic, they sound boomier.”

Headphones, used almost invariably by participants during NMP, also had a positive effect on feelings of intimacy. Dominic explains:

“You end up with a more intimate experience or a more intimate sound than if you were there in person… When I'm in person, I'm at least a few feet away. I'm not right on top of the person's face because that's rude. After using the low latency stuff, I was like, Oh, this is actually clearer! I can hear my conductor more clearly on low latency than in person because in person, she would be 10 feet away with a high ceiling, there would be a lot of bouncing echoing and stuff. And with (NMP) it's just like we're all climbing on top of each other's shoulders and muttering directly into each other's ears.”

Dominic's account shows the paradox of intimacy at a distance in sharp relief: the remote setup can produce a form of auditory proximity unavailable in ordinary physical space. Yet not everyone found this desirable. Jordan noted her ensemble mates' preferences for playing in person, when they could hear the “music flying around the room, being able to feel it, vs. it being here,” as she pointed to her headphones. “I mean, I don't like it either,” she added. “I prefer not to wear headphones on stage.” The intimacy allowed by studio sound tools, then, is a specific condition, appreciated by some, resisted by others, and clearly different from the conditions of co-located music performance.

#### Safety and convenience

Participants also connected intimacy at a distance to the safety, convenience, and comfort of remote participation. As remote work became normalized in other sectors during and after the COVID-19 pandemic, similar benefits appeared in participants' descriptions of NMP. Daphna summarized these advantages directly: “Nobody has to drive—they don't have to commute. You don't have to schlep your equipment… So it's super, super convenient.” Convenience was not incidental; for some participants, it shaped the conditions under which they could make music at all. Jocelyn, who is immunocompromised and has not performed live since 2019, described turning down an invitation to sing in person: “I told her that I don't think I can perform, because I would have to wear a special mask to sing… I could probably leave the doors open, but I still feel very unsafe… For me, (NMP) is the way I play with people (now).” Her account underscores that for some musicians, the medium was not simply an alternative. It had become a primary means of sustaining musical connections and community.

Several participants explicitly described NMP as comfortable. “It feels very, very comfortable,” said Joan of her regular practice. John described helping others manage incoming sound in ways that contributed to both comfort and intimacy:

“You can lower or raise the volume at which you receive the other person, which is a key feature for your comfort, and you can apply dynamic compression (to smooth out the volume received). So you can be comfortable and not feel like your (ears are being) abused. But I think musically, it's a lot of intimacy.”

The experience of intimacy at a distance unfolded within and partly because of conditions of safety, comfort, and environmental control.

#### Global connections

If telepresence, studio-quality sound, and safety and convenience all shaped the conditions of intimacy at a distance, a sense of shared global connections was often the consequence. Participants repeatedly described meaningful musical relationships forged across countries, continents, and the accompanying large latencies. Bella, for example, engaged in music making from Sweden with collaborators in Brazil and India. With thousands of miles between them, no shared first language, and signal latency equivalent to sound traveling through air from opposite ends of a football field, the trio nonetheless formed meaningful artistic and personal bonds. As Bella explained, “You can be in different parts (of the world) and still be together in a way and communicate. That is extremely enjoyable.”

For some participants, the magnitude of the distance changed how latency was perceived. Rather than serving primarily as a source of frustration, it could become part of the accepted condition of connection. Joan described an audience response after an international performance:

“I interviewed the audience afterwards, and I asked, ‘Did you notice that latency? The delay?' And they're like, ‘What? What delay?' Like they, didn't even *know* that there was a delay. And it seemed almost like an insulting question (to imply) that somehow something was out of sync… because it felt like such a synchronous experience, why would I even imply that something was wrong?”

Bill described a similar attitude regarding a connection between Scotland and New Zealand: “Given that we were so geographically far apart, it almost didn't matter. I just gave myself over to accepting (the latency) as it was, and just tried to enjoy the process.” In these accounts, the global connections forged were more important than experiencing temporal immediacy.

Others, however, remained committed to steady-beat music even in international contexts. Declan described piano duos between Paris and New York: “We really got good at figuring out what tempos worked, what tempos didn't.” He also described a continent-spanning European band whose singer lived in Senegal. Because the singer was delayed more than the instrumentalists but could “float like crazy,” the arrangement still worked. Declan's account points to the liminal space between synchrony and asynchrony.

Participants did not experience synchrony as a simple binary, but as a field of nuance, role-specific flexibility, and stylistic possibility. Jordan summarized her experience of intimacy at a distance with a nod to science fiction:

“I don't have to say anything in English for them to understand the music. That's what I like about (NMP): I can connect to these other artists in distant places. And we can create together. We can blend our musical aesthetics, our personalities. Our identities can connect. It's the closest thing we have right now to a teleportation device.”

Independently, Kristin used similar language when describing the felt experience of terminating an NMP session: “I have left a space! It really does feel like I have very abruptly left a place. It's as close to teleporting as anything I can imagine.” Across participant accounts, intimacy at a distance names a recurring experiential pattern: participants felt close while distanced and connected while delayed.

### Theme 2: improvisation

The dynamic and sometimes unpredictable nature of NMP rewards a high level of improvisational skill from its participants. Findings related to the second theme of improvisation show how musicians adapted to and innovated within the constraints and opportunities afforded by NMP. Through adaptive strategies and creative musical practices, participants navigated latency and distance to create meaningful and cohesive musical experiences.

#### Adaptive strategies

Participants showed a high degree of flexibility. Adaptive strategies included revising one's concept of “real time” to accommodate high and/or variable latency, thinking of latency as “back phrasing” (the idiomatic jazz practice of intentionally and dynamically delaying one's singing or playing), and lowering performance tempi.

One prominent adaptive strategy involved reconceptualizing “real time.” Participants often displayed an intuitive sense of the phenomenological slipperiness of time. Whether this grew out of NMP or led them toward it is difficult to say, but an elastic concept of time clearly opened a wider range of possible practices in latency-rich settings. When I suggested to Rachel that for some musicians the essence of music is mastery of synchronous playing through real-time negotiation of pulse and pitch, she objected strongly to my use of the term “real time.” “It *is* real time,” she insisted. “You are performing in real time. It's just at a distance.” For Rachel, the proposition that latency might invalidate a musical practice was offensive. She described the experience of NMP as “real time in different spacetime,” calling it “phenomenologically a really interesting experience.” Her account makes clear that for some participants, the challenge was not only technical but conceptual: how to understand shared musical time when participants are experiencing the effects of mediated practice.

Joan likewise emphasized temporal plurality: “I'm not exclusionary toward steady beat, but in my own aesthetics, I'm much more oriented to (the idea of a) time network… I've tried to open up lots of different ideas about time, because time is very malleable.” Her emphasis on malleability helped make sense of a medium in which participants were often operating with different local experiences of timing and delay.

Participants also described revamping musical practice itself. John came up with a strategy to relocate his own perception of latency by using an input latency compensator in the NMP software Sonobus. Instead of hearing latency between his piano and the remote singers, he preferred to experience the delay between his own physical action and the sound returning to him:

“Sonobus has a feature that it copied from Jamulus in which you can hear yourself with (a delay equivalent to) the server latency… So when I do that, as an electric pianist, I can isolate myself from the outside world and just use headphones. And you know, it sounds later, but you hear it simultaneously, the voice of the singer and your piano. It's actually way less cognitive load to adapt your *playing* than to adapt your *hearing*.”

For John, the tradeoff was worth it because it aligned his monitoring more closely with the ensemble sound. His adaptation was bodily and perceptual at once.

Participants also described tempo strategies. Dominic recounted the rhythmic bodily effort required to maintain tempo when fellow singers seemed to drag: “Hold. The. Tempo! Hold. The. Tempo!” Otherwise, he said, “it will do the death spiral thing (of getting progressively slower).” John independently used the same “death spiral” phrase to describe a situation in which singers were told to follow John's piano so that the tempo would not slow. He called this type of musical sacrifice swallowing the latency: “It's just going to sound horrible for me. I just need to not hear (the others) very much… There's an element of sacrifice, an element of service from the accompanist who needs to swallow the latency.”

Others used slower tempos or less rhythmically demanding repertoire. Beau described how a slow, rhythmically open duet with his brother succeeded partly because “it's a slow piece. It's sort of free. There's no rhythm to it, really.” Declan described finding “the right tempos” for remote piano duos between Paris and New York. These remarks suggest that adaptation often took the form of selecting repertoire and tempos suited to the latency conditions at hand.

#### Musical creativity

NMP both engendered musical creativity and attracted the musically creative. The same spirit of improvisation that stimulated the adaptive strategies described above also manifested as jazz improvisation, avant-garde improvisation, electroacoustic experimental music, and intermedia arts. The creative practices recounted by some participants suggest an expanded definition of musicking, organized around temporal relation as much as sound. Rather than using the word “music,” Joan preferred to think of the practice as “time-based arts,” with many expressive possibilities emerging precisely because “time is such a fundamental factor of this network setting.”

Among other participants who preferred to play steady beat music, latency remained a problem to solve, or else to work carefully around. Declan described a session with about 125 ms of delay and said, “I wouldn't want to be doing that all the time. Because it really takes away that immediacy, (which is) one of the things that is most wonderful about music.” Kristin similarly described successful efforts to support jazz students at her higher education institution during the COVID-19 pandemic, proudly stating that they “played fast music” and were not prevented by latency from “making beautiful music,” and that furthermore they were able to “continue their regular ensemble courses in a time when almost nobody was able to do that.”

By contrast, practitioners of avant-garde or open improvisation often treated latency not as a problem to be minimized but as an integral part of the experience. Carter noted the longstanding association between telematics and improvisation, invoking figures such as George Lewis and Pauline Oliveros. Jordan described a trio performance that combined prerecorded samples and live improvisation, then lamented how few musicians in academia are taught “avant-garde experimental music improvisation.” Bella described an international project across three continents built from live audio, drone instruments, percussion, whisper, and voice, structured through rehearsal but without sheet music. As she explained, the piece became “a collaborative structure” or “instant composition,” requiring people who knew enough about composition, improvisation, listening, and response to develop motives into larger forms together.

Intermedia arts extended these creative orientations beyond sound alone. Joan highlighted the term “intermedia” as opposed to the more common “multimedia” because NMP involves different art forms working together and responding to the same gesture “within their respective media.” For example, Simon described a project in which a live installation video performance using Isadora software interacted with his own sound work, the video being “audio reactive.” Such examples show that NMP was often lived not simply as remote ensemble playing, but as a broader exploratory field encompassing multiple media, sensory modes, and performance logics.

### Theme 3: practical hope for the medium

For the purposes of the present paper, “practical hope” means a lived orientation toward actionable possibility under uncertain or unstable conditions. “Practical hope for the medium” therefore names participants' approach to NMP as a technically contingent, socially contested, but musically meaningful practice. Participants demonstrated practical hope for the medium by seeking the best possible tools, advocating for NMP, and rebutting skepticism about its value.

#### Seeking out the right tools

For many, the genesis of their NMP practice was asking a question along the lines of: *What softwares exist that allow for low-latency music performance?* Participants were also discriminating about their choice of headphones, microphones, interfaces, computers, connectivity, and ancillary software. They discussed digital audio workstations and specialized digital tools for electroacoustic, improvisational, and experimental music. Their accounts show a willingness to pursue complex or demanding technical setups in order to realize the medium's possibilities (and their own artistic intentions) more fully.

Isaac, for example, described his broader efforts to teach music students to use professional tools at a professional level: “Don't just teach them how to make a recording with Audacity; teach them how to make a recording with Logic Pro… The solution cannot be ‘We're just going to have them use GarageBand on their phones.”' Though not about NMP *per se*, Isaac's remarks exemplify the progressive, technology-forward disposition many participants brought to their practice.

John described similar concerns from a different economic and infrastructural context. At the beginning of the pandemic, he tried to recruit others into NMP but found that the necessary tools could be prohibitively expensive where he lived. “You really need good equipment,” he said, and that was “a limiting factor in so many countries.” He described people asking him for quotes on NMP kits (microphone, USB audio interface, Ethernet connections, etc.) and then recoiling at the price. John's account shows that the search for appropriate tools was not merely a matter of preference. It was shaped by uneven economic conditions that made the professional-grade tools Isaac assumed accessible to some but not others.

#### Proselytizing

Participants often described a felt need not only to use NMP, but to persuade others of its value. Kristin referred to the NMP community as a “secret society” and to the people willing to try it out as her “adventure pals.” She explained: “I feel more kinship with a person who's willing to do this intrepid experience with me… So like, my relationships with the people that I make music with online are inherently different than with anyone else.” In her account, willingness to try the medium functioned as both a social bond and a threshold of belonging. When asked who had been most receptive to trying NMP, Kristin answered with a broad smile, “Oh, the youths!” She described how, in higher education settings, students initially required to use the tools came to recognize the medium's value for themselves. At the same time, she acknowledged the awkwardness of trying to bring others in: “The way that we have to evangelize it in order to get people to join feels cultish.” Her language suggests that participation in NMP often involved not just explanation, but a kind of conversion work aimed at overcoming skepticism, unfamiliarity, or reluctance.

Declan likewise stressed that NMP had to be experienced, not merely described: “I think it's really interesting that this is a kind of superpower, that when people experience it, pretty universally their mind is blown. And yet, most people—including most musicians—still don't know this is possible.” His account captures the testimonial quality that ran through many participants' descriptions: because the medium was widely unknown or misunderstood, committed users often felt compelled to demonstrate it, advocate for it, and initiate others into its possibilities.

#### Confronting skepticism

Participants frequently expressed frustration at what they saw as a combination of inexperience, ignorance, and skepticism toward NMP. Isaac paraphrased a dismissive response to low-latency music making he perceived from many academics, musicians, and technologists: “They're basically like: ‘It's all snake oil, and it can't possibly work!”' He attributed such reactions less to malice than to profound unfamiliarity with the medium. Kristin described the distress of doubt in the midst of demonstrable success: “I cannot tell you how gaslighting (and) distressing it was to be in the room making music with folks at a very high level, and then have 99% of the internet be like ‘This is impossible!'.” For Kristin, the mismatch between direct experience and widespread disbelief has remained significant even in the years after the COVID-19 pandemic, with many people still assuming that music performance in an online milieu must always lead to an impoverished quality of experience.

Others confronted skepticism more futuristically. Carter described a dinner conversation with people from a large technology corporation who asserted that worldwide latency could never be improved beyond a certain point. He responded not by denying the absolute nature of the speed of light, but by speculating about predictive engines similar to those used in online gaming:

“What if we can synthesize your partner in real time here on our side, ahead of what they do but also *guided* by what they do, so it's a prediction engine? How far ahead does it have to be? 100 ms? Is that so far out of reach?”

Whether or not the algorithmic prediction tools Carter imagines become viable, his account—along with those of Kristin, Daphna, Declan, John, and others—captures the forward-driving character of the third and final theme of practical hope for the medium. Participants did not simply use available tools. They imagined, argued for, and anticipated new ones. Practical hope for the medium was a lived orientation toward a personally valid musical practice, requiring various combinations of curiosity, advocacy, onboarding, and technical experimentation.

#### Essence of the phenomenon

The central essence of the lived experience of NMP was a felt sense of expanded musical possibility across physical distance. Participants described inhabiting a medium in which connection, creativity, and shared presence remained available despite latency, separation, and technological mediation. Intimacy at a distance, improvisation, and practical hope for the medium were all consistent features of the phenomenon. Intimacy at a distance named the paradoxical experience of feeling close while geographically dispersed, shaped by telepresence, studio-quality sound, domestic comfort, and global connectivity. Improvisation described both the adaptive strategies and the creative orientations through which participants negotiated latency, revisited temporal assumptions, and made musically viable use of the medium's constraints and affordances. Practical hope for the medium captured participants' lived orientation toward NMP as a fragile but actionable musical possibility, requiring tool-seeking, recruitment, and confronting skepticism. Taken together, these themes suggest that NMP was lived as an expanded field of possibility for connecting, collaborating, and creating with others across distance in the age of digital networks.

## Discussion

Actor-Network Theory (ANT), as developed by Latour (1987, 2005) and further informed for use in this study by the musicologist Born (2005); Born and Barry (2018), provides a theoretical framework for understanding the interactions that typify Networked Music Performance (NMP). Central to ANT is the recognition that objects, too, participate in the action. If we call technologies “just tools,” we risk black boxing their role; in many cases, the tool demonstrably mediates, constrains, and redistributes action (Latour, 2005). A metronome, for example, does not merely extend a musician's will; it reorganizes temporal attention, entrains bodily behavior, and can even override a performer's internal sense of pulse (Repp, 2005). With the phenomenon of NMP, the network of actors extends beyond the musicians to include musical instruments, audio signal processors, musical source material, microphones, headphones, analog-to-digital and digital-to-analog converters, software, routers, and the larger network infrastructures through which the data travel.

ANT also holds that local and large-scale forces jointly assemble an experience (Latour, 2005). For example, a compressor setting, a microphone's response curve, the distance between continents, and the quality of regional infrastructure are all capable of shaping the same musical moment. NMP thus makes unusually palpable the continuity between intimate musical interaction and wider infrastructural conditions. Extending Latour's work, Born and Barry (2018) explain that music's existence is always constituted by some combination of sound, embodied practices, discursive interpretations, visual inscriptions, material devices, commodity forms, physical location, venue, and social relations. This view maps closely onto the themes developed from participants' accounts in the present study.

In co-located music performance, certain mediations recede into familiarity, while in NMP, the same are impossible to ignore. In a club or concert hall, musical connection is ordinarily achieved through both conscious and subconscious alignments of sound, space, gesture, attention, proximity, and timing. NMP practitioners, by contrast, are obligated to think carefully about latency, routing, headphone mix, microphone response, and software settings because these mediating factors enter the musical event in undeniable ways. NMP thus renders unusually visible the conditions through which musical connection is ordinarily achieved. Actor-Network Theory sharpens the visibility of these conditions in the following discussion of themes.

### Actor-networks and intimacy at a distance

The theme of intimacy at a distance is especially well suited to ANT because it names a form of musical connection produced through heterogeneous mediations rather than through physical co-presence. In participants' accounts, NMP did not foreclose feelings of intimacy, even when thousands of miles of distance—and the accompanying latency—separated performers. What appears at first as a paradox of closeness across separation becomes, in Born's (2005) terms, an instance of music's status as the “paradigmatic multiply-mediated, immaterial and material, fluid quasiobject, in which subjects and objects collide and intermingle.” (p. 7). Intimacy in NMP, then, is not simply preserved despite mediation; it is assembled through the very relations that redistribute nearness, distance, comfort, and shared musical presence. Following Latour and Born, each subtheme below names a mediation by pairing two mutually informing dimensions (spatio-temporal; technical-acoustic; domestic-bodily; infrastructural-social).

First, the subtheme of telepresence can be understood through ANT as mediated spatio-temporal relation. In music, shared space and shared time are ordinarily intertwined: performers coordinate through acoustic vibrations, bodily gestures, visual attention, and the felt immediacy of sounding together. In NMP, those relations are redistributed through networked conditions, creating both inherent limitations to practice and the potential for otherwise impossible musical events. Kristin's description of having “left a space” after a session, Isaac's habit of imagining a room proportioned to the measured milliseconds of delay, and Carter's creation of digitally modeled acoustic environments all suggest that NMP reassembles musical space through altered temporal relations and likewise reassembles musical time through altered spatial relations.

The subtheme of studio-quality sound points toward a second mode of mediation: technical-acoustic mediation. This mediation includes not only microphones, headphones, interfaces, signal paths, and monitoring practices, but also the continual conversion of acoustic sound into data and vice versa. In NMP, intimacy was often experienced through this chain as a heightened sonic nearness made possible by audio technologies. For some participants, that intimacy lay in the unusual clarity, detail, and proximity of the sound itself. Dominic's image of musicians “muttering directly into each other's ears” captures this especially well. At the same time, participants such as Jordan reminded us that the spaciousness and diffusion of co-located sound in a room could be preferable to the closeness afforded by headphone-based listening. In Gibson's (1979) ecological sense, headphone-based listening alters the *affordances* of ensemble sound, affecting what participants could hear, monitor, balance, and experience as intimate. Identified in these accounts was a form of intimacy assembled through technical-acoustic mediation that could heighten connection for some musicians while producing discomfort or aesthetic loss for others.

The subtheme of safety and convenience reveals a third mode of mediation: domestic-bodily mediation. Participants' descriptions make clear that intimacy at a distance unfolded within the practical realities of home, health, fatigue, and control over one's immediate environment. Jocelyn's account is especially important here, since NMP had become the primary means through which she could continue making music with others at all. For Daphna and others, the absence of commuting, equipment transport, and other logistical burdens altered the conditions under which musical participation was possible. Participants also described comfort in relation to volume control, dynamic compression, and the ability to regulate incoming sound. These accounts suggest that intimacy at a distance was shaped partly by domestic-bodily mediations that made participation feel manageable and safe.

Global connectivity introduces a fourth mode of mediation: infrastructural-social mediation. Participants described meaningful relationships forged across countries, continents, languages, and temporal displacement. One striking feature of participants' descriptions was the way local and large-scale conditions entered the same lived moment together. A headphone mix, a microphone's response curve, the distance between continents, and the quality of regional infrastructure were all capable of shaping the same musical event. NMP thus made unusually palpable the continuity between intimate musical interaction and wider infrastructural conditions.

Taken together, these views of how mediation can manifest help clarify why intimacy at a distance was so central to the findings. Participants were describing a mode of connection assembled across multiple layers. Feelings of intimacy were neither uniform nor unlimited. Quality of experience varied with latency, fidelity, repertoire, role, trust, and technical setup. Yet for many advanced users it was nevertheless vivid and artistically satisfying.

### Actor-networks and improvisation

Whether through musical, technological, or procedural improvisations, participants showed remarkable flexibility in response to latency, unpredictability, and other demands of network-mediated interaction. They broadened what it means to be performing together in “real time,” reconceptualized latency as back phrasing in jazz contexts, adjusted tempo ranges, manipulated pan and volume settings, and used latency as a limiting condition that in turn generated creativity. Participants adjusted their habits, their expectations, and sometimes their internal sense of time itself.

In ANT, social structures are the result of ongoing interactions and negotiations among diverse actors. Participants' general penchant for improvisation therefore engendered—even *created*—the requisite conditions for nonlocal performance networks to emerge. NMP also requires musicians to persevere toward artistic ends with ever-changing constellations of tools, signal paths, partners, roles, and affordances. Findings suggest that advanced users are particularly skilled at acting within those shifting networks. Improvisation thus extends beyond musical content to include the ongoing maintenance of a viable performance network.

The prevalence of jazz improvisation in this study is illuminated through ANT's focus on fluid and dynamic interactions. The ontology of jazz resists the older modernist notion that value is located primarily within the static score. Kane (2014) has noted how well jazz maps onto ANT, observing that when musicians, singers, lyricists, arrangers, and improvisers rework songs, they open a black box and begin to fiddle with its parts. This inclination to “fiddle with” the medium recurs throughout the data, including Rachel's array of audio-processing tools, John's problem-solving around latency compensation, and Declan's relentless efforts to update software with new features. Whether through divergent thinking, musical improvisation, or technical adjustment, these advanced users of NMP tended to value exploration and contingency.

Findings also suggest a meaningful divergence between musicians who pursue the feel of co-located synchrony and those who treat latency as integral to telematic art. Jazz musicians in the study often sought latency levels low enough to preserve rhythmic immediacy and groove (i.e., below 30 ms). Open improvisers and telematic artists more readily embraced quasisynchrony, distributed attention, and temporal looseness as realities of the medium. Whether latency was minimized or embraced, improvisation remained central to how participants inhabited NMP as a musically workable environment.

### Actor-networks and practical hope for the medium

Participants sought tools, recruited and onboarded others, and confronted skepticism because NMP appeared to them as both possible and precarious. Dominic's immersive headset microphone, Rachel's signal-processing practices, and Joan's interest in multichannel hybrid performance all demonstrate participants' efforts to make NMP's possibilities materially available rather than merely imaginable. Viewed through ANT, this hope for the future of NMP was an active force in the assembly and maintenance of the network. Participants sought to assemble the conditions under which their emergent practice could become visible, audible, credible, and repeatable. Practical hope for the medium therefore named not only a participant orientation, but one of the practices through which the network maintained and extended itself.

### Future directions

Several future directions emerge directly from these findings. First, research should continue to examine the divergence between musicians who approach NMP as an approximation of co-located synchrony and those who approach it as a medium with its own aesthetic priorities and measures of success. That distinction appeared repeatedly across interviews and seemed to shape how participants evaluated latency, repertoire, risk, and even the meaning of “real time.” Comparative work across a range of NMP ensembles could clarify when low-latency immediacy is experienced as essential, when quasisynchrony is acceptable, and when temporal looseness becomes artistically generative. Such inquiry could prove consequential for NMP software design, repertoire choice, and the language through which NMP is described, assessed, and taught.

Second, future research should investigate the experiential effects of mediation more directly. Participants described intermittent telepresence, heightened sonic intimacy, domestic comfort, distributed attention, and, in some cases, an abrupt sense of disconnection when sessions ended. These reports suggest a need for further qualitative and mixed-methods work on the phenomena of presence, attention, fatigue, trust, and social bonding in the context of NMP. Studies that pair phenomenological interviews with event-level technical data—latency, jitter, packet loss, routing configurations, and acoustic features—could help clarify how specific material conditions shape the felt quality of connection.

Third, more work is needed on the material and infrastructural conditions of NMP. If one contribution of this study is to show how nonhuman actors can mediate musical experience, then those actors should themselves become objects of inquiry. Comparative studies across levels of equipment quality and network stability could help determine which conditions most strongly affect musical coordination, perceived intimacy, artistic satisfaction, and ease of use. The future of NMP will also depend on more than technical innovation. It will also depend on who can afford participation, who has reliable access to suitable infrastructure, and which musicians stand to benefit most—or be excluded—from the medium's continued development.

Finally, future studies should continue to explore NMP across distinct use cases. Private lessons, chamber rehearsals, collaborative songwriting, composition, jazz improvisation, and intermedia performance all place different demands on timing, communication, embodiment, and evaluation. Comparative inquiry across such use cases could clarify how different performance ecologies organize musical relation. Participants' accounts also suggest that continued work by developers to lower latency, offer multichannel sound, and simulate three-dimensional environments may alter the phenomenology of the medium in significant ways. The the central question here concerns how such changes reshape presence, relation, creativity, and the forms of musicking that networked environments make possible.

## Conclusion

The purpose of this study was to understand the nature of the experience of Networked Music Performance for advanced users. Findings suggest that these practitioners did not experience network mediation exclusively as an obstacle to be overcome. Instead, many treated NMP as a unique milieu to be approached on its own terms. Intimacy emerged through headphones, microphones, latency, and physical distance—conditions often assumed to weaken musical connection. “Real time” appeared not as a fixed threshold but as an elastic experiential category, negotiated through latency compensation, tempo adjustment, back phrasing, repertoire choice, and the acceptance of multiple simultaneous presents. This elasticity contributed to the centrality of improvisation, which participants described both as creative musical practice and as a broader strategy for negotiating unpredictable network conditions. Participants consistently demonstrated practical hope for the medium, enacted through tool-seeking, recruitment, and resistance to skepticism about NMP's musical viability. NMP was therefore lived not as an impoverished substitute for co-located performance, but as a distinctive field of mediated artistic possibility.

Participants' accounts further suggest that NMP reorganizes musical interaction, leading to new affordances and constraints. For some, the guiding goal of NMP was to reproduce the immediacy of co-located synchrony. For others, latency and distributed attention opened new aesthetic and ontological possibilities for their artistic practice. Across these differences, the present study points toward a temporal ecology in which multiple presents can coexist and meaningful musical relation can be sustained under the conditions of network mediation. In this sense, NMP does not dissolve distance so much as teach musicians new ways of inhabiting it.

What this study finally reveals is not the disappearance of mediation, but its experiential thickening. Networking tools and their consequent conditions all entered directly into the felt texture of musical experience. Future work will no doubt trace these conditions with greater technical precision, but the present findings already suggest that networked musicking has become a lived terrain rather than a speculative frontier. In Latourian terms, the question is no longer whether relation can survive mediation, but how mediation reshapes what relation feels like. The significance of NMP, then, lies not only in extending music across space, but in rendering newly perceptible the conditions through which musical togetherness is assembled at all.

## Data Availability

The raw data supporting the conclusions of this article will be made available by the authors, without undue reservation.
